# An Unusual Case of Obstructive Jaundice Secondary to Pyogenic Liver Abscesses

**DOI:** 10.7759/cureus.16409

**Published:** 2021-07-15

**Authors:** Shannon K Brewer, Pranav Patel, Riad Kesiry

**Affiliations:** 1 Internal Medicine, University of North Texas Health Science Center, Fort Worth, USA; 2 Internal Medicine, Medical City Weatherford, Weatherford, USA

**Keywords:** pyogenic abscess, liver abscess, obstructive jaundice, cholestasis, liver abscess aspiration

## Abstract

Pyogenic liver abscesses (PLA) are an uncommon, but potentially life-threatening infection. Although the link between amebic liver abscesses and obstructive jaundice is well documented, there are few cases of PLA leading to this complication. We present a case of multiple massive PLA and obstructive jaundice on initial presentation. The patient was treated for six weeks with antibiotics and percutaneous drains placed in the largest abscesses and was discharged after clinical improvement and resolution of the hyperbilirubinemia. This case highlights the importance of clinician awareness of other etiologies when evaluating patients with signs and symptoms of painful obstructive jaundice.

## Introduction

The formation of hepatic abscesses secondary to bacteria has been well established; however, extensive disease-causing obstructive jaundice is rare. Pyogenic liver abscesses (PLA) are uncommon, but account for approximately 80% of all hepatic abscesses [1] and are associated with significant rates of mortality. The most common causative organisms of PLA include *Streptococcus anginosus* group organisms anaerobes, and gram-negative bacteria including *Escherichia coli* and *Klebsiella pneumoniae* [2]. Obstructive jaundice is a rare manifestation of PLA. Here we present a case of multiple massive PLA presenting as obstructive jaundice in an immunocompetent patient.

## Case presentation

A 48-year-old Hispanic male with no significant past medical history presented with four days of right upper quadrant abdominal pain and vomiting. Upon presentation, he was febrile and tachycardic. Physical examination was notable for right upper quadrant abdominal tenderness, hepatomegaly, and jaundice. He denied any recent travel, alcohol use, or illicit drug use.

Labs were significant for leukocytosis of 27.67 x 10^3^/µL with neutrophilic predominance and lactic acid 2.9 mmol/L. The results of hepatic-function tests suggested obstructive jaundice with total bilirubin 14.0 mg/dL, direct bilirubin 11.35 mg/dL, aspartate aminotransferase 388 U/L, alanine aminotransferase 271 U/L, and alkaline phosphatase 480 µ/L. Blood cultures revealed *S. constellatus* bacteremia. Transthoracic echocardiogram was unremarkable. Extensive evaluation for underlying malignancy or other predisposing infectious etiologies, including carcinoembryonic antigen, cancer antigen 19-9, serum alpha-fetoprotein tumor markers, hepatitis, and HIV serologies, was negative.

**Figure 1:**
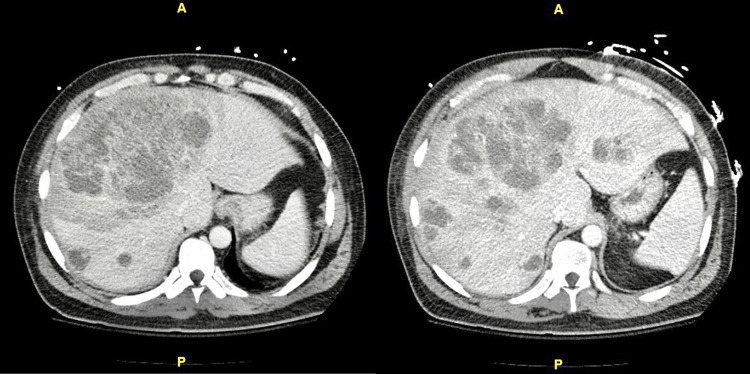
Computed tomography of the abdomen showing innumerable low-density hepatic lesions throughout with the largest complex lesion centered about the right lobe measuring 12 cm x 16 cm.

**Figure 2:**
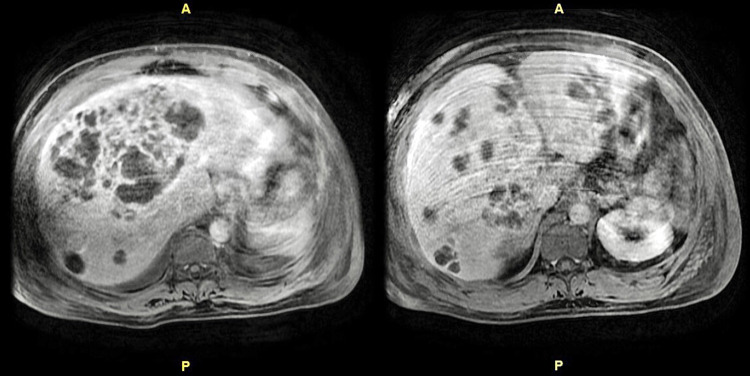
Triple-phase magnetic resonance imaging of the abdomen redemonstrating innumerable hepatic abscesses with the largest lesion demonstrating phlegmon-like features.

Abdominal ultrasound showed multiple hepatic masses. Computed tomography (CT) of the abdomen revealed marked hepatomegaly and innumerable low-density masses throughout the liver with a large complex mass in the right lobe measuring up to 12 cm x 16 cm (Figure 1), redemonstrated by triple-phase magnetic resonance imaging (MRI) of the abdomen (Figure 2). Cultures from liver abscess aspiration revealed *S. intermedius*.

The patient was treated with broad-spectrum antibiotics and two percutaneous drains were placed in the largest abscesses, producing a significant amount of drainage with considerable clinical improvement and subsequent removal of the drains after four weeks. He was discharged and completed six weeks of daptomycin without further incidence.

## Discussion

PLA are rare, occurring in 2.3 cases per 100,000 hospital admissions [3], but account for the majority of hepatic abscesses. The most common causative organisms isolated in PLA cultures were *E. coli* (32%) and *K. pneumoniae* (45%) [4]. Other common organisms include *S. aureus* (20%), *Streptococcus* species (3.4-15.5%), and polymicrobial infections (7.6-79%) [5,6]. Sources of PLA include cryptogenic (56%), biliary (15.5%), intraabdominal sources via the portal vein (15%), hematogenous (13.5%) [4,7], direct extension from localized disease, and trauma [8]. Patients with immune deficiency, malignancy, sickle cell anemia, diabetes mellitus, and liver transplant have a high risk of developing a liver abscess [9]. Mortality associated with PLA is approximately 30% despite appropriate treatment and can be as high as 80-100% if left untreated [7]. Concurrent malignancy, abscess multilocation, hemoglobin less than 10 g/dL, total bilirubin greater than 1.05 mg/dL, and failure of percutaneous drainage are risk factors associated with higher mortality rates. Notably, mortality is higher in patients with multiple liver abscesses compared to those with a solitary lesion [7,10].

Multiple abscesses account for approximately 33% of cases [9], are of biliary origin (45%), and often associated with *E. coli,* whereas single abscesses are typically cryptogenic (58.9%) and often associated with *K. pneumoniae* [10]. Mortality rates in patients with multiple liver abscesses and single liver abscesses are 22.1% and 12.8%, respectively [7,10].

Traditionally, PLA were treated with surgical drainage and broad-spectrum antibiotics. However, with the advent of minimally invasive procedures, the gold standard has shifted to ultrasound or CT-guided percutaneous intervention alongside culture-guided antibiotic therapy [4,8]. However, primary surgical intervention is still recommended in cases with abscess rupture, multiloculated abscesses, biliary communication, or inadequate percutaneous drainage [8].

Hepatic abscesses have a classic triad of clinical syndromes that include right upper quadrant pain, fever, and jaundice that is only seen in 10% of patients [7]. Hyperbilirubinemia is a relatively uncommon presentation in PLA with elevated total serum bilirubin levels noted in 12.5-36.2% of cases [4,11].

The pathophysiology of obstructive jaundice secondary to PLA is likely due to compression and distortion of the biliary tree by the abscesses, causing intrahepatic cholestasis. Multiple and single large amoebic liver abscesses on the inferior surface near the porta hepatitis were seen most often in cases of hepatic duct compression in patients with jaundice [12-14]. Similar pathophysiology was seen in several cases of patients with polycystic liver disease [15,16].

Benign causes of obstructive jaundice include choledocholithiasis (62.5%), biliary strictures (25%), and chronic pancreatitis (6.3%) [17]. Malignant causes include carcinoma of the gallbladder (28.7%), carcinoma of the pancreas (26.5%), and cholangio-carcinoma (10.8%) [18]. Obstructive jaundice secondary to PLAs is exceedingly rare with few documented cases in the literature [19,20]. However, other cases of obstructive jaundice secondary to amebic liver abscesses have been well established [12,14]. Cholestasis with hyperbilirubinemia is present in 29% of amebic liver abscess cases and levels of serum bilirubin are directly correlated to the size and number of abscesses [12]. Although mild jaundice is a common finding in patients with amebic liver abscess, severe obstructive jaundice is a rare complication [18]. In cases of amebic liver abscesses, jaundice is observed in 8-22% of patients [13]. Additionally, patients with obstructive jaundice have increased mortality when compared with non-jaundiced patients undergoing surgical procedures, thus emphasizing the importance of early detection [17].

## Conclusions

This case illustrates a unique presentation of obstructive jaundice secondary to pyogenic hepatic abscesses. This complication of PLA is rare and, to our knowledge, there are only two other similar cases in the literature. This case highlights the importance of clinician awareness of other etiologies when evaluating patients with signs and symptoms of painful obstructive jaundice.
